# Supplementary material for: Podocyte-Related Mechanisms Underlying Survival Benefit of Long-Term Angiotensin Receptor Blocker
Source: Int J Mol Sci. 2022 May 27;23(11):6018. doi: 10.3390/ijms23116018 (PMC9181646; doi:10.3390/ijms23116018)
Supplement: Supplementary file 1 [file ijms-23-06018-s001.zip › ijms-1720933-supplementary.pdf]

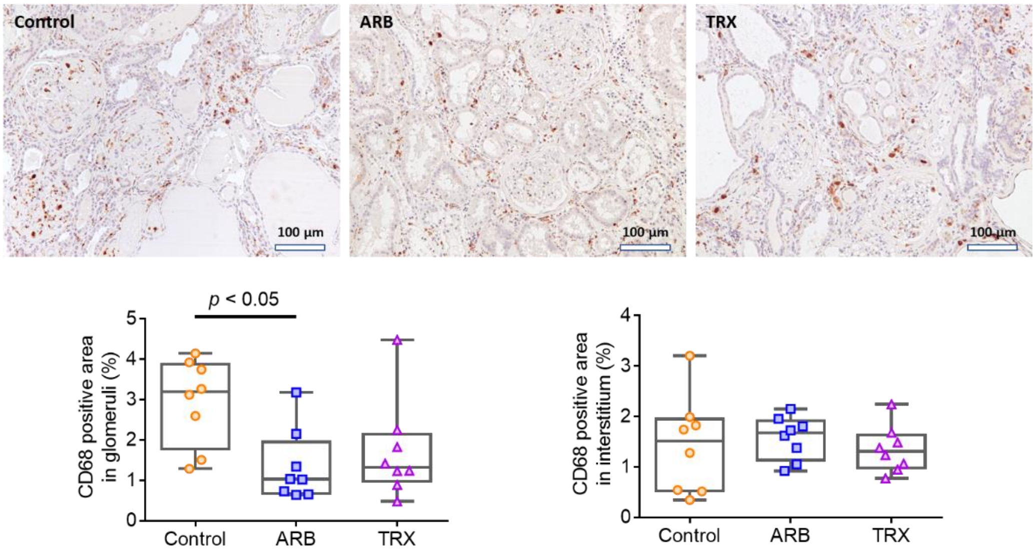

Figure S1. Long-term ARB and TRX treatment and renal inflammation. ARB significantly and TRX numerically reduced macrophage infiltration in glomeruli vs Control, while there was no different interstitial inflammation among three groups (X200).
